# Novel Variants in *PUS7* Associated With Intellectual Disability and Growth Retardation: Expanding the Clinical Spectrum in 13 Patients

**DOI:** 10.1111/cge.70192

**Published:** 2026-06-05

**Authors:** Camille Bergès, Clément Sauvestre, Sophie Naudion, Catherine Vincent Delorme, Thomas Smol, Mélanie Rama, Stephanie Moortgat, Isabelle Maystadt, Rogier Kersseboom, Martina Wilke, Tahsin Stefan Barakat, Yoann Vial, Laurence Perrin, Sandrine Passemard, Farooq Ahmad, Muhammad Umair, Tobias Haack, Mona Grimmel, Alma Kuechler, Anne Slavotinek, Patrick Devine, Ugur Hodoglugil, Faisal Zafar, Erum Afzal, Tracy Dudding‐Byth, Guillaume Jouret, Lucas Herissant, Stephanie Efthymiou, Henry Houlden, Marine Legendre, Vincent Michaud

**Affiliations:** ^1^ Service de génétique médicale, CHU Bordeaux, MRGM‐U1211, INSERM/Université de Bordeaux Bordeaux France; ^2^ CHU Lille, Institut de Génétique Médicale, Université de Lille Lille France; ^3^ Centre de Génétique Humaine, Institut de Pathologie et de Génétique Gosselies Belgium; ^4^ Center for Heritable Syndromes Zuidwester Middelharnis the Netherlands; ^5^ Department of Clinical Genetics Erasmus University Medical Center Rotterdam the Netherlands; ^6^ Department of Genetics Robert DEBRE University Hospital, Sorbonne‐Paris‐Cité University, INSERM‐UMR‐1141 Paris France; ^7^ Department of Chemistry Women University Swabi Swabi Pakistan; ^8^ Medical Genomics Research Department King Saud Bin Abdulaziz University for Health Science Riyadh Saudi Arabia; ^9^ Department of Life Sciences University of Management and Technology Lahore Pakistan; ^10^ Institute of Medical Genetics and Applied Genomics University of Tübingen Tübingen Germany; ^11^ Institute of Human Genetics University Hospital Essen Essen Germany; ^12^ Division of Medical Genetics University of California, San Francisco San Francisco California USA; ^13^ Genomic Medicine Laboratory University of California, San Francisco San Francisco California USA; ^14^ Department of Development Pediatrics The Children's Hospital Multan Pakistan; ^15^ University of Newcastle Callaghan New South Wales Australia; ^16^ National Center of Genetics LNS Dudelange Luxembourg; ^17^ Department of Neuromuscular Diseases University College London London UK

**Keywords:** aggressiveness, genetics, growth retardation, neurodevelopment, PUS7, sequencing

## Abstract

Pseudouridylation is a frequent post‐transcriptional modification resulting in uridine isomerization in 5‐ribosyluracil, also called pseudouridine. This mechanism leads to RNA stability with an increase in base‐stacking and the creation of hydrogen bonds. Recently, papers reported that variants in *PUS7* in 16 patients were involved in marked growth retardation with microcephaly, associated with intellectual disability and behavioral issues such as self‐injurious and aggressive behavior. Through *Genematcher*, we initiated a collaboration to describe a new cohort of *PUS7* patients. In total, we report 13 new cases carrying 15 new variants. This cohort further expands the phenotypic spectrum associated with *PUS7*‐related syndromes, allowing for improved genotype–phenotype correlations and ultimately better healthcare for affected individuals and their families.

## Graphical abstract

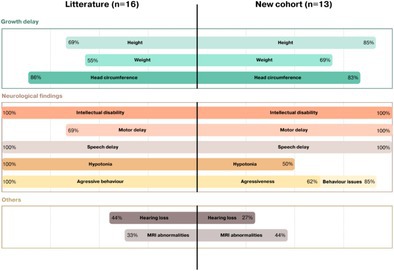

## 1 Introduction

Many post‐transcriptional RNA modifications have been described the past years, ranging from adenyl methylation (m^6^A),—the most abundant internal modification—to pseudouridylation, the most common RNA modification found in cells [1, 2]. These modifications, which play roles in many physiological processes, have also been associated with different pathologies.

Pseudouridylation is the isomerization of the Uracil into 5‐ribosyluracil, known as Pseudouridine (Ψ) or the “fifth RNA nucleotide” [3]. This isomerization is carried out by a class of enzymes called pseudouridine synthases (PUS). In humans, 13 pseudouridine synthases have been described. Given its distinct chemical properties compared to uridine—particularly the extra hydrogen bound donor–Ψ can stabilize Ψ‐A base pairs through base stacking and water coordination. Therefore, this results in an increase in the thermal stability of the RNA and a greater rigidity of the nucleotide backbone, thereby influencing RNA stability and secondary structure. Physiologically, Ψ has been associated with elevated protein expression and is believed to play a direct role in splicing and translation [4, 5].

Misregulation of protein translation is implicated in many human pathologies, and certain syndromes have been associated with loss of PUS activity. Pathogenic variants in *PUS1* and *PUS3* have been described in recessive inherited syndromes with affected patients presenting with growth delay, secondary microcephaly, intellectual disability, and seizures [6]. These pseudouridylate synthases defects and their impact on RNA structure (especially tRNA) were validated in vitro, establishing the first connections between pseudouridylation pathways and mendelian monogenic diseases. Similarly, *PUS7* variants have also been described in patients with growth deficiency, microcephaly, intellectual disability, and abnormal behaviour, especially aggressiveness [7, 8, 9, 10, 11]. Despite overlapping clinical features, the clinical spectrum of these syndromes remains poorly defined. Moreover, the rarity of reported cases complicates efforts to understand the underlying pathophysiology. Han et al. proposed that the loss of PUS7 activity in these individuals dysregulates protein translation, leading to increased MYC protein levels and decreased HPRT1 protein levels without impacting the mRNA levels of either gene. This imbalance may impair cellular differentiation through MYC dysregulation, while the neurological features and failure to thrive is proposed to result from the HPRT1 misregulation, resembling a Lesch–Nyhan syndrome‐like.

Here, we report 13 new cases, expanding the phenotypic spectrum of *PUS7*‐related syndrome.

## 2 Methods

*PUS7* variants were identified by laboratories using targeted gene panels, exome sequencing, or whole genome sequencing. Details about the sequencing strategy for each patient can be found in Supporting Information. Variants were retrieved through a worldwide collaborative network via GeneMatcher. Variants are annotated according to the NM_019042.5 transcript and classified regarding the guidelines of the American College of Medical Genetics and Genomics. Population frequencies are based on gnomAD v4.1 version. Missense and splicing predictions were assessed using standard in silico tools available on Mobidetails (CADD, AlphaMissense, SpliceAI, and SPiP, see supplemental references). Effects on splicing were analysed with functional validation performed through cDNA analysis from fibroblasts obtained via skin biopsy for P1 and P10 (Figure S1). Informed consent was obtained from the patients and/or their parents before genetic analysis was performed. ConSurf was used to assess evolutionary conservation; scores were visualized as a heatmap using the ggplot2 package in R. 3D protein in silico comparison between wild type WT and variant was performed using DynaMut2.

## 3 Results

### 3.1 Literature Overview

**FIGURE 1:**
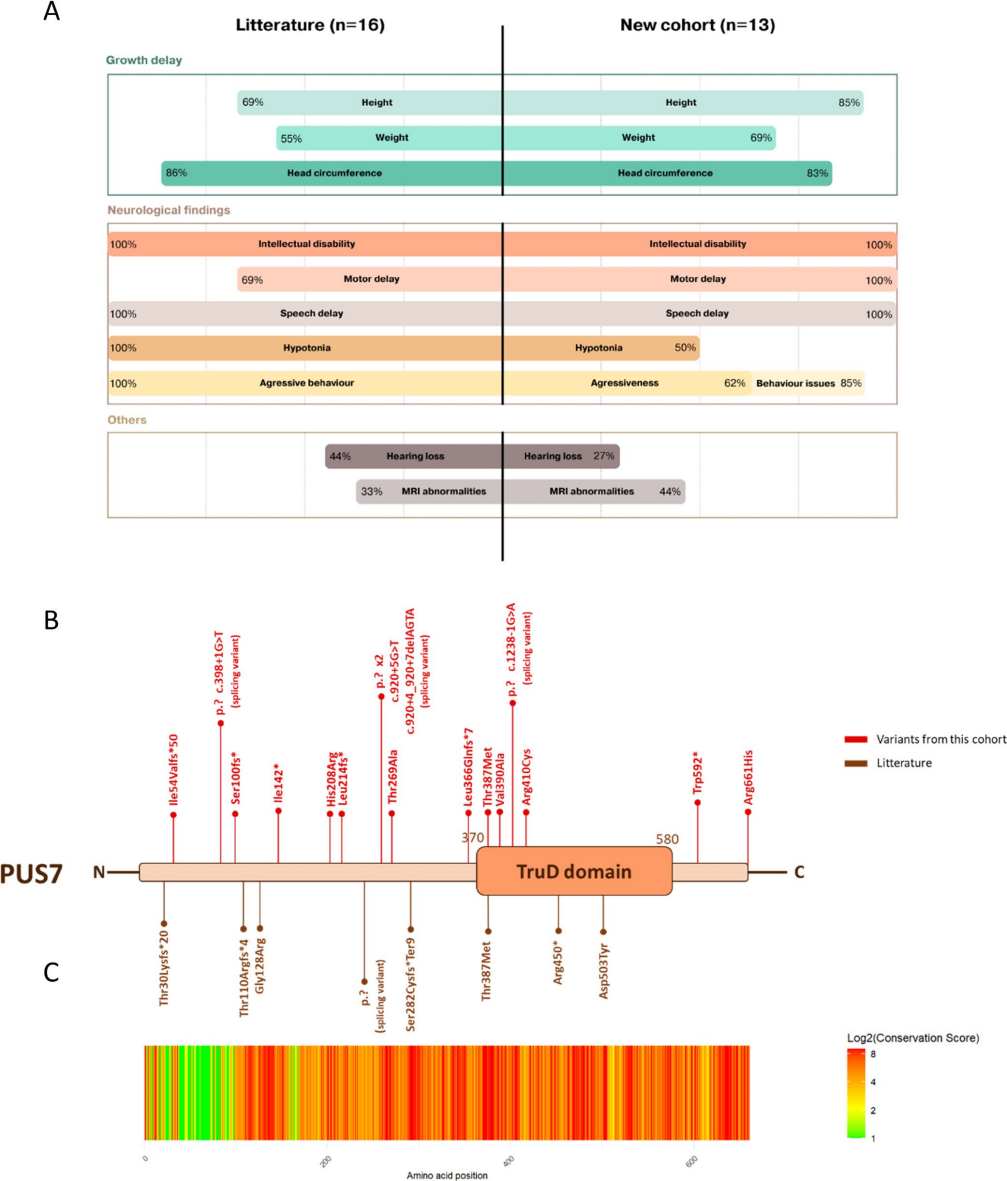
Clinical and molecular overview of previously reported and new PUS7 patients. (A) Clinical features represented in percent compared to the existing literature. (B) New PUS7 variants repartition through the protein. (C) Amino acid conservation within PUS7 domains. The conservation score is a log_2_ representation of ConSurf results that scale from 1 to 9 (most conserved).

In previously published cohorts [7, 8, 9, 10, 11, 12], several clinical features appeared to be constantly observed in patients with *PUS7* variants. (Figure 1; Table S1). Regarding the growth delay, 86% of patients had microcephaly, 69% showed short stature and 55% presented with low body weight. At the neurological level, 100% patients exhibited intellectual disability and a speech delay. Additionally, 100% of the patients displayed behavioral issues, with aggressiveness being a prominent feature. Motor delay was observed in 69% of cases. Some dysmorphic features were also reported but most were inconsistent, with the exception of a smooth philtrum, which was present in 85%. Hearing loss was noted in four individuals. The genetic alterations described were mainly homozygous events, resulting in nonsense variants. Three missenses were described, two of which were located within the TruD catalytic domain of PUS7.

### 3.2 Clinical Description of Our Cohort

In this new cohort, we report 13 additional patients who share phenotypic features with the previously described cases. Growth delay was a consistent finding, with 85% exhibiting short stature and 69% having low body weight. Microcephaly was present in 83% of individuals, with a mean head circumference of approximately −2.68 SD (Figure 1; Table 1). All the patients had intellectual disability with a motor delay and a marked speech delay. Hypotonia was observed in 42% of cases. The majority exhibited behavioral issues, with 62% showing auto or hetero‐aggressive behavior. Interestingly, three of the newly described patients had hearing loss. Brain MRI abnormalities were identified in 50% of cases, but no specific pattern could be observed. Importantly, we were able to collect clinical data from adult individuals, the oldest being 23 years old. This provided valuable insight into the long‐term prognosis of *PUS7*‐related syndrome. These older patients appeared to show stable symptoms over time, with no evidence of a progressive or degenerative course.

**TABLE 1:** New cohort clinical features.

	P1	P2	P3	P4	P5	P6	P7
Age at last examination	6 years	9 years	6 years	23 years	8 years	6 years	12 years
Gender	Female	Female	Female	Female	Female	Female	Male
Height	**−4.3 SD**	**−3.3 SD**	0 SD	**−2 SD**	**−3.4 SD**	**−2.99 SD**	**−2.2 SD**
Weight	**−3.2 SD**	**−2.8 SD**	−0.34 DS	**−2 SD**	**−3.08 SD**	**−2.93 SD**	**−2.8 SD**
HC	**−2.8 SD**	**−3.8 SD**	−1 SD	**−2.7 SD**	**−4.4 SD**	**−2.05 SD**	**−3.9 SD**
Intellectual disability	+	+	+	++	++	+	+
Motor delay	+	+	+	+	+	+	+
Speech delay	+	+	+	+	+	+	+
Hypotonia	−	−	+	+	+	−	−
Behavioral problems	**Aggressive**	Not aggressive, feeding issues	Behavioral issues unspecified	**Aggressive** behavior	**Aggressive**/auto‐aggressive behavior, hyperactivity	Aggressive behavior not noticed; May have sensory integration disorder	**Aggressive**
Skeletal	Normal	Normal	5th clinodactyly			Normal	Normal
			Costal histiocytosis				
Other	Dental malposition, repeated otitis, cutaneous xerosis	MRI: Loss of cerebral volume and delayed degree of myelination for the patient's age, Thin corpus callosum	Short stature at birth. with – 2 SD in all parameters	Hypergonadotropic hypogonadism	Normal MRI	**Moderate bilateral hearing loss**	MRI: reduced white matter
	Normal MRI		Normal MRI			MRI: Thin corpus callosum	

	P8	P9	P10	P11	P12 (brother of P11)	P13	Total
Age at last examination	2 years	20 years	2 years	22 years	17 years	7 years	
Gender	Male	Male	Male	Male	Male	Female	
Height	**−2.2 SD**	**−3.68 SD**	**−2 SD**	**−4.5 SD**	**−5.5 SD**	+1.82 SD	11/13–85%
Weight	−1 SD	−0.27 SD	**−3.7 SD**	**−5 SD**	**−4.5 SD**	+4.93 SD	9/13–69%
HC	−**1.9 SD**	NK	**−5.1 SD**	**−2.5 SD**	**−2.5 SD**	−0.7 SD	10/12–83%
Intellectual disability	+	+	+	++	++	+	13/13–100%
Motor delay	+	+	++	+	+	+	13/13–100%
Speech delay	+	+	+	+	+	+	13/13–100%
Hypotonia	−	−	+	+	+	−	6/12–50%
Behavioral problems	**Aggressive**, Stereotypies, isolation, auto stimulation	Stereotypies. shivers when excited, hypersensitivity to loud noises (mowers and vacuums)	No	manual stereotypies.ASD, self‐injurious behavior, **aggressiveness**	Manual stereotypies.ASD, self‐injurious behavior, **aggressiveness**	Stereotypies, **auto and heteroaggressive** behavior, eating disorder, frustration intolerance	11/13 (85%) with 8/13 displaying aggressive behavior (62%)
Skeletal	Clinodactyly	Normal		Scoliosis	Scoliosis		
				Bilateral radio unlar synostosis	Bilateral radio unlar synostosis		
Other	**Hearing Loss**	**Mild conductive hearing loss**		Normal MRI	Normal MRI		3/11 hearing loss—27%
	MRI: Small hypophyse						4/9 MRI abnormalities—44%

*Note:* Bold values hilghlight out of range features.

Abbreviations: HC, head circumference; MRI, magnetic resonance imaging; NK, not known; SD, standard deviation.

### 3.3 Molecular Findings of Our Cohort

The new reported patients predominantly carried homozygous variants, often due to consanguinity. Only P1, P6, P8, P10, and P12 presented with compound heterozygous variants. With the exception of the splicing variant c.398+1G>T (P13), the deletion of exon 15 (P11, P12), and the missense p.(Thr387Met) (P1), all variants are novel and have not been previously reported (Figure 1B; Table 2). All newly identified variants were rare (absent or 0 homozygous occurrences in control database GnomAD v4.1). Truncating variants were identified in patients P4, P5, P6, P8, P10, P11, and P12. Variants with potential splicing effects were also identified. In P1 and P8, the c.920+5G>T and c.920+4_920+7delAGTA variants appear to affect exon 7 splicing, with SpliceAI Donor Loss scores of 0.99 and 1, respectively, indicating a probable loss of the 5′ splicing donor site. This effect was confirmed by RNA‐seq in P1 (Figure S1), which showed exon 7 skipping in nearly 50% of the reads. Exon 7 skipping preserves the reading frame, but this deletion results in the loss of 26 amino acids within the TruD catalytic domain. In P10, the c.1238‐1G>A variant is predicted to disrupt exon 11 splicing, potentially leading to a loss of the 3′ acceptor site (SpliceAI AL = 0.99, SPiP risk score = 98.41%), that could either result in exon 11 skipping or the use of an alternative acceptor site at c.1238–9 (SpliceAI AG = 0.61). RNA‐Seq confirmed exon 11 skipping in 68% of reads but not the use of an alternative acceptor site. This patient is compound heterozygous for another variant located in exon 3 c.424del. Analysis by RNA‐Seq shows that this variant causes an intron retention which was not predicted by in silico scores (Figure S1). Missense variants were also identified. The C‐terminal domain is highly conserved (Figure 1C), suggesting a critical role in PUS7 function. This hypothesis is further supported by the missense variant found in P3 p.(Arg661His), which is located in this terminal region of the protein (Figure 1B). P1 variant p.(Thr387Met) is located within the TruD catalytic domain. In silico predictions classify it as deleterious (see Table 2 for all in silico predictions). Interestingly, this variant was previously reported by Han et al. and western blot analysis confirmed that it disrupts PUS7 activity, with an increased rate of protein translation in patient fibroblasts compared to controls [8]. Additional missense variants within the highly conserved TruD catalytic domain (Figure 1A,B) were found in P2 and P7: p.(Val390Ala) and p.(Arg410Cys) were both predicted deleterious by in silico tools. The last reported missenses for P9 p.(Thr269Ala) and P13 p.(His208Arg) do not occur within a known functional domain of PUS7. However, each receives strong in silico likely pathogenic predictions. For p.(Thr269Ala), the deleterious predictions are likely due to its putative impact on PUS7 structure, with a predicted ΔΔG^Stability^ of −1.21 kcal/mol according to DynaMut2 (Figure S2). In the same way, p.(His208Arg) for P13 has a predicted destabilization of the protein structure (ΔΔG^Stability^ predicted = −0.68 kcal/mol; Figure S2). A limitation of this study is the absence of direct functional validation for the new missense variants reported. While in silico predictions and structural modelling provide supportive evidence, functional assays such as pseudouridylation sequencing (Ψ‐seq) or enzymatic activity measurements would be necessary to definitively assess the impact of these variants on PUS7 activity.

**TABLE 2:** New cohort molecular features.

	P1		P2	P3	P4		P5	P6	
						
Ethnicity	European caucasian		South asian	European caucasian	European caucasian		Libanon	Asian	
Consanguinity	No		**Yes**	No	No		**Yes**	No	
Zygosity	Compound Heterozygous		Homozygous	Homozygous	Compound heterozygous		Homozygous	Compound heterozygous	
Sequencing method	WES trio		WES + Sanger sequencing for probands	WES duo	WES trio		WES Trio	WES trio	
Segregation	Paternal	Maternal	Inherited from each parent	Mother htz	Paternal	Maternal	Inherited from each parent	Paternal	Maternal
				Father unkown					
Nucleotide change	c.1160C>T	c.920 + 5G>T	c.1169 T>C	c.1982G>A	c.1263C>A	c.96delA	c.1097_1098del	c.298_299delAG	c.640_641delCT
Type of variation	Missense	Splicing	Missense	Missense	**Nonsense**	**Frameshift**	**Frameshift**	**Frameshift**	**Frameshift**
Protein change	p.Thr387Met	p.? (Splicing impact)	p.Val390Ala	p.Arg661His	p.Cys421*	p.Lys32Asnf*9	p.Leu366Glnfs*Ter7	p.Ser100fs*	p.Leu214fs*
Population frequency (GnomAD v4.1)	22 Heterozygotes	Absent	1 Heterozygote	135 Heterozygotes	7 Heterozygotes	Absent	Absent	11 Heterozygotes	6 Heterozygotes
	0 Homozygote		0 Homozygote	0 Homozygote	0 Homozygous			0 Homozygote	0 Homozygote
Predictions	CADD Phred 29.3	Splice AI DL 0.96	CADD Phred 26.7	CADD Phred 26.70	—	—	—	—	—
	AlphaMissense 0.985 Likely Pathogenic	SPICE 0.99 high	AlphaMissense 0.955 Likely Pathogenic	AlphaMissense 0.141 Likely benign					
		SPiP Risk score 98.41%							
			
ACMG Classification	Class 5	Class 4	Class 4	Class 3	Class 5	Class 5	Class 5	Class 5	Class 5
	Pathogenic	Likely pathogenic	Likely pathogenic	VUS	Pathogenic	Pathogenic	Pathogenic	Pathogenic	Pathogenic
	*PS1, PS3, PS4, PM2, PM3, PP4*	*PS3, PM2, PM3, PP4*	*PM1, PM2, PP3, PP4*	*PM2, PP3, PP4*	*PVS1, PM2, PM3, PP4*	*PVS1, PM2, PM3 PP4*	*PVS1, PM2, PP4*	*PVS1, PM2, PP4*	*PVS1, PM2, PP4*

	P7	P8		P9	P10		P11	P13	
							P12 (brother of P11)		
Ethnicity	Saraiki Pakistani	European caucasian		European caucasian	Mauritian		Moroccan	European caucasian	
Consanguinity	**Yes**	No		**Yes**	**Yes**		**Yes**	No	
Zygosity	Homozygous	Compound heterozygous		Homozygous	Compound heterozygous		Homozygous	Compound heterozygous	
Sequencing method	WES + Sanger sequencing for probands	WES Trio		WES trio	WGS Trio		WES Trio	WES trio	
Segregation	Inherited from each parent	Paternal	Maternal	Inherited from each parent	Maternal	Paternal	Inherited from each parent	Paternal	Maternal
Nucleotide change	c.1228C>T	c.920 + 4_920 + 7delAGTA	c.156_159delGTCC	c.805A>G	c.1238‐1G>A	c.424del	Exon 15 deletion	c.398 + 1G>T	c.623A>G
Type of variation	Missense	Splicing	**Frameshift**	Missense	Splicing	**Nonsense**	**Nonsense**	Splicing	Missense
Protein change	p.Arg410Cys	p.? (Splicing impact)	p.Ile54Valfs*50	p.Thr269Ala	p.? (Splicing impact)	p.Ile142*	p.Trp592*	p.? (Splicing impact)	p.His208Arg
Population frequency (GnomAD v4.1)	24 Heterozygotes	Absent	Absent	3 Heterozygotes	Absent	Absent	Absent (+Absent from DGV)	1 Heterozygote	50 Heterozygotes
	0 Homozygote			0 Homozygote				0 Homozygote	0 Homozygote
Predictions	CADD Phred 34	SpliceAI DL 1	—	CADD phred 26.10		—	—	SpliceAI DL 0.98	
	AlphaMissense 0.963 Likely Pathogenic	SpliceAI DG 0.31		AlphaMissense 0.995 Damaging	SpliceAI AL 0.99			SpliceAI DG 0.26	CADD Phred 26.90
		SPiP Risk score 98.41%			SpliceAI AG 0.61			SPiP Risk score 98.41%	AlphaMissense 0.994 Likely Pathogenic
					SPiP Risk score 98.41%				
ACMG Classification	Class 4	Class 4	Class 5	Class 3	Class 4	Class 5	Class 5	Class 4	Class 4
	Likely pathogenic	Likely pathogenic	Pathogenic	VUS	Likely pathogenic	Pathogenic	Pathogenic	Likely pathogenic	Likely pathogenic
	*PM1, PM2, PP3, PP4*	*PM2, PM3, PP3, PP4*	*PVS1, PM2, PP4*	*PM2, PP3, PP4*	*PP1, PS3, PM2, PP4*	*PVS1, PS3 PM2, PP4*	*PVS1, PM2, PP4*	*PM2, PP3, PS4*	*PM2, PP3, PS4*

*Note:* Bold values hilghlight consanguinity and truncating variant.

Abbreviations: htz, heterozygous; NK, not known.

## 4 Conclusions

Thanks to next‐generation sequencing technologies, RNA modifications have been extensively studied in recent years, revealing their critical role in many biological processes including cell differentiation, sex determination and stress responses. Consequently, RNA modifications have been implicated in a wide range of diseases—from Mendelian disorders to cancer [13]. Those RNA modifications are catalysed by RNA “erasers” such as demethylases, pseudouridylate synthases, methyltransferases etc. Pseudouridylate synthases 1, 3 and 7 deficiencies have been linked to developmental disorders that share overlapping phenotypes, particularly intellectual disability and growth delay [7, 8, 9, 10, 11, 12, 14]. In this study, we report 13 new cases of *PUS7*‐associated syndrome, including 15 newly identified variants, significantly expanding the number of documented cases in the literature. This work confirms and further refines the clinical spectrum of *PUS7*‐related syndrome. Notably, intellectual disability associated with speech delay is observed in 100% of the individuals. Behavioral issues, particularly aggressiveness, are reported in the majority of patients. Severe growth delay, especially microcephaly, represents a prominent recurrent clinical feature. Interestingly, hearing loss was observed in 27% of cases, supporting previous findings suggesting that this could be a related symptom. Skeletal signs were only described for two patients' siblings (P11 and P12); it will be important to screen future patients for these features. This newly described cohort enhances our understanding of the clinical spectrum of *PUS7*‐related syndrome, and contributes to improved genetic counselling and follow‐up for affected families. Importantly, no clear genotype–phenotype correlation could be established. Most of the newly identified variants were truncating variants leading to a premature stop in PUS7, encompassing the TruD catalytic domain, as previously reported in the literature. Additionally, we identified new missense and splicing variants, which will require further functional studies to confirm their effective pathogenicity.

The role of RNA regulation through pseudouridylate synthases is not completely understood. Given the overlapping clinical features observed in patients with *PUS1*, *PUS3*, and *PUS7* deficiency, it would be relevant to investigate whether those proteins could have a redundant role in different cell types. According to the GTEx database, all three proteins are ubiquitously expressed. Investigating their specific RNA targets and potential compensatory mechanisms could provide key insights into their respective roles and the variability of phenotypic expression in patients.

The growing body of evidence supporting the involvement of RNA post‐transcriptional modifications in human diseases highlights the emerging relevance of epitranscriptomic analyses in clinical settings. In this context, investigating epitranscriptomic profiles in patient‐derived samples could represent a valuable tool for variant interpretation and disease modelling. These approaches have been extensively tested in cancer, shedding light on specific epitranscriptomic signatures [11]. Epitranscriptomic profiles can be performed using LC–MS/MS approaches, but recent advances in next‐generation sequencing have enabled transcriptome‐wide mapping of modified nucleosides. In the case of pseudouridylate synthases, Ψseq, a Ψ‐specific Chem‐Seq can be done. It would be interesting to investigate whether genome‐wide Ψ‐seq profiles exhibit specific patterns in *PUS7*‐deficient patients.

## Author Contributions

C.B. and V.M. compiled the molecular and clinical data. All authors contributed to clinical and molecular data and reviewed the manuscript. C.B. performed the conservation and 3D modelling analyses. C.B. and V.M. conceived, coordinated and supervised the study. C.B. and V.M. wrote the main manuscript and prepared the figures.

## Funding

RNA‐Seq for P1 was part of the RID project funded by the GIRCI SOHO and the University Hospital of Bordeaux (CHUBX 2021/40; Clinical trials NCT05696912). Research reported in this publication was supported by the National Human Genome Research Institute at the National Institutes of Health under Award Number U01HG009599. The content is solely the responsibility of the authors and does not necessarily represent the official views of the National Institutes of Health.

## Ethics Statement

This study was approved by the Comité de Protection des Personnes Sud Ouest et Outre Mer III and was conducted in accordance with the tenets of the Declaration of Helsinki. Written informed consent was obtained from all individuals before genetic analysis. Skin biopsy for patient P1 is part of a clinical research project (RID CHUBX 2021/40; Clinical trials NCT05696912) approved by the Comité de Protection des Personnes Ile de France VII (2022‐A00918‐35). Authorization for publication was obtained from the participants.

## Conflicts of Interest

The authors declare no conflicts of interest.

## Supporting information


**Figure S1:** Aberrant *PUS7* splicing in P1 and P10 fibroblast RNA‐seq data. Visualization is done thanks to Sashimi plot tool on IGV. This transcript analysis is compared to two control RNA from WT PUS7 patients. (a) P1 RNA‐seq: an exon 7 skipping is observed in 10 reads/26. Other events are visualizable, including a possible intronic retention that need to be better characterize. (b) P10 RNA‐seq: c.1238‐1G variant is involved in an exon 11 skipping, with 24/35 reads. The c.424del variant is also involved in an intron retention which was not predicted with in silico scores (SPiP and Splice AI).

**Figure S2:** In silico 3D protein modelling of P9 and P13 variants. (a) The c.805A>C p.(Thr269Ala) variant is represented according to DynaMut2^20^. ΔΔG^Stability^ predicted stability change is −1.21 kcal/mol. (b) The c.623A>G p.(His208Arg) variant is represented according to DynaMut2^20^. ΔΔG^Stability^ predicted stability change is −0.68 kcal/mol. Purple dotted lines represent clash interactions, orange dotted lines represent polar bonds, yellow dotted lines represent ionic interactions, light blue dotted lines represent Van der Walls interactions, green dotted lines represent hydrophobic interactions.

**Table S1:** PUS7 syndrome, literature overview. HC, head circumference; hmz, homozygote; N, normal; NR, not reported; SD, standard deviation; MRI, magnetic resonance imaging.

**Data S1:** Supplemental data: Resume of clinical presentation and sequencing strategy for patients included in the study.

## Data Availability

The data that support the findings of this study are available from the corresponding author upon reasonable request. Variants were uploaded to the Clinvar public database.
