# Supplementary material for: Novel Variants in PUS7 Associated With Intellectual Disability and Growth Retardation: Expanding the Clinical Spectrum in 13 Patients
Source: Clin Genet. 2026 Jun 5;110(3):379–88. doi: 10.1111/cge.70192 (PMC13431721; doi:10.1111/cge.70192)
Supplement: Supplementary file 4 — Data S1: Supplemental data: Resume of clinical presentation and sequencing strategy for patients included in the study. [file CGE-110-379-s003.docx]

**Supplemental data : Resume of clinical presentation and sequencing strategy for patients included in the study.**

**Patient 1** is a 6 years old girl, the first child of a non-consanguineous unaffected couple. Intra-uterine growth deficiency was noted during pregnancy. Feeding difficulties were noted at 4 months old. She presented with global development delay and aggressive behavior. Examination found staturoponderal delay as well as microcephaly. Previous genetic testing consisted of karyotype and array-CGH. Trio exome sequencing was performed with Sure Select (Agilent) capture kit and sequenced on HiSeq4000 platform (Illumina) by Integragen (details about filtration strategy can be found in Angelini et al. EJMG, 2021 PMID: 33667650).

**Patient 2** is a 9 years old girl. She presented with global development delay and staturoponderal delay as well as microcephaly. Single whole exome sequencing found a homozygous likely pathogenic missense variant in *PUS7* that was confirmed by sanger sequencing (details about filtration strategy can be found in Khan et al. Mov Disord, 2021 PMID: 34820905).

**Patient 3** is a 6 years old girl. Intra-uterine growth deficiency was noted during pregnancy. Developmental delay was found on global and fine motor skills and on speech. Examination found strabismus, myopia, blepharophymosis, hypertelorism, ptosis, full cheeks, microstomia, short philtrum, and clinodactyly of the fifth digits. Duo exome sequencing was performed using Illumina DNA Prep Enrichment 150and bp paired-end exome sequencing was performed on an Illumina NovaSeq 6000 platform, with an average on-target coverage of 150X and analyzed using GATK best practices. The variants were annotated using Variant Effect Predictor in combination with the in-house pipeline ANATOLE2.

**Patient 4** is a 23 years old female patient with severe intellectual disability and autism. During the first months, feeding was difficult and she had recurrent vomiting. Transition from milk to solids was very difficult. She was hypersensitive in the oral area. Her motor development was delayed. She could walk by 24 months. She has never had speech, but can understand simple sentences. The patient has several dysmorphic features, microcephaly, small stature, deep set eyes, periorbital fullness, anteverted nares, broad philtrum, narrow palate and small nails on hand and feet, with a sandal gap. Neurological examination reveals generalized hypotonia and a broad-based gait. Patient has been diagnosed in the past with hypergonadotropic hypogonadism. Previous investigations included amongst other an unremarkable metabolic screening in serum and urine, a normal karyotype, a normal SNP-array, normal Fragile X testing. Upon trio whole exome sequencing (performed as previously described Deng et al., Acta Neuropathol 2023 PMID: 37119330), two *PUS7* compound heterozygotes variants were found c.1263C>A/p.Cys421* and c.96delA/p.Lys32Asnfs*9.

**Patient 5** is an 8 years old girl. She presented with global development delay and aggressive behavior. She has also staturoponderal delay and microcephaly. *PUS7* variant was identified via trio exome sequencing (Agilent Sure Select Human All Exon V6), which was performed on an Illumina system (NovaSeq6000). The variant was also validated by Sanger sequencing in both the index patient and the parents. For details regarding variant calling, filtering and bioinformatics, see reference Fritzen et al., Hum Genet. 2018 PMID: 29796876.

**Patient 6** is a 6 years old girl. She presented with global development delay and aggressive behavior. She has also staturoponderal delay and microcephaly. Whole exome sequencing was performed using xGen Whole Exome Panel kit (Integrated DNA Technologies) on a HiSeq 2500 device (Illumina) and found two compound heterozygous *PUS7* variants (c.298_299delAG/p.Ser100Phefs*11 and c.640_641delCT/p.Leu214Valfs*11). More details about filtering strategies can be found in Slavotinek et al., NPJ Genom Med. 2023 PMID: 37236975.

**Patient 7** a 12-year-old boy from a consanguineous family of Saraiki Pakistani descent (parents are second cousins). He is the oldest of seven children in the family. There was history of 1 IUD and 1 miscarriage. The pregnancy of the boy was at 8 months and delivery was IVD. The boy presented at the age of 3 years with generalized tonic seizures. EEG examination in the boy was abnormal due to diffuse slowing of background activity. He was administered Epival and the frequency of the seizures is mild to moderate. He also presents with delayed developmental milestones with isolated motor delay, poor speech development which includes slurring and stammering, as well as delay to sit, walk or thrive and moderate intellectual disability. He also presents with strabismus. Growth parameters for the boy at 12 years of age was 48cm head circumference (-3.9SD), height of 133cm (-2.2SD) and weight of 24 kg (-2.8SD). Other clinical features included aggression and raised ammonia and lactate on biochemistry tests. Brain MRI imaging done at yes at 8 years of age showed reduced white matter suggestive of infective ischemic brain. Simplex exome sequencing identified a homozygous *PUS7* missense variant (c.1228C>T/p.Arg410Cys). Detailed methodology of exome sequencing can be found in Mencacci et al., Am J Hum Genet 2016 PMID: 27058447. Sanger sequencing of his parents confirmed heterozygosity for this variant in each of the parents.

**Patient 8** is a two years old boy. Family history is negative with a non-affected twin brother. He presented with speech and motor delay (sitting and standing but no independent walking). Bilateral moderate deafness was noted. Previous genetic testing included array-CGH and Fragile-X. Trio whole sequencing revealed two compound heterozygous pathogenic variants. Exome sequencing was done with Sure Select Human All Exon V8 (Agilent) capture kit and sequenced on NextSeq550Dx platform (Illumina) (details about filtration strategy can be found in Angelini et al. EJMG, 2021 PMID: 33667650).

**Patient 9** is a 20 years old male presenting with severe intellectual disability, short stature and autistic traits. Examination found some dysmorphic features: long nose, short philtrum, wide nasal tip and thin upper lip. Trio exome sequencing was done upon Mendeliome v1.159 panel app targets and revealed a homozygous *PUS7* variant c.805A>G, p.(Thr269Ala).

**Patient 10** is a two years male presenting with short stature, microcephaly and developmental delay. Trio whole genome was performed on Seqoia national sequencing platform with NEBNext® Ultra II library preparation and sequenced on NovaSeq 6000 platform (Illumina). Detail about filtering strategy can be found in Tusseau et al., Eur J Med Genet 2024 PMID: 38355093.

**Patients 11 and 12** are two young boys aged 7 and 2, originated from Morocco whose parents are first cousins. Both have severe global delays in psychomotor development, progressive microcephaly, and short stature (-5 SD for one and -2 DS for the younger). The older boy has developed scoliosis. Dysmorphic features include a flat face and long philtrum in the younger boy. They have low-set ears and a thin upper lip, as well as limited supination of both forearms, suggesting radio-ulnar synostosis, which was confirmed by standard X ray. All these clinical characteristics led us to consider a diagnosis of Tsukahara syndrome. Fifteen years later, they are 22 and 17 years old and have developed severe ID with a total lack of autonomy in daily life, absence of language, significant motor agitation, and a very complicated behavioral picture with ASD, aggression, anxiety, anger and self-harm, especially in the older child which has also sialorrhea. Microcephaly is stable (-2,1/-3 SD) and both have significant failure to thrive (Weight, - 3,5 SD; Height: -5 SD). Genetic analysis consisted of trio whole exome sequencing revealing *PUS7* variant (see filtering strategy in Moortgat et al., Hum Mutat 2021 PMID: 33942450).

**Patient 13** is a 7-year-old female born at 37 weeks and 6 days of gestation after an unremarkable pregnancy. Birth parameters were within the normal range, with a birth weight of 3130 g, length of 50 cm, and head circumference of 35 cm. At the last evaluation, she presented with early-onset obesity, with a weight of 50 kg (+4.93 SD) and a height of 132 cm (+1.82 SD); head circumference was 50.5 cm (−0.7 SD). Neurodevelopmental assessment revealed moderate intellectual disability with global developmental delay. Independent walking was achieved at 24 months. Speech was markedly limited, consisting of isolated words without sentence formation. Behavioral abnormalities included stereotypies with hand-flapping, heteroaggressivity, and autoaggressivity. Severe feeding disturbances were reported, characterized by absence of satiety and food-seeking behavior. Sleep disturbances were also noted, including difficulties initiating sleep and obstructive sleep apnea. Craniofacial examination revealed a long philtrum, epicanthus, downslanting palpebral fissures, short palpebral fissures, full lips, and an everted lower lip. Additional investigations did not reveal recurrent infections, organ malformations, or abnormalities on cardiac or abdominal ultrasound. Given the syndromic association of neurodevelopmental disorder, behavioral abnormalities, and early-onset obesity, a genetic etiology was suspected. Therefore, trio whole-genome sequencing (WGS) was prescribed in order to investigate a potential monogenic cause. Genomic DNA was extracted from EDTA anticoagulated peripheral blood using the QIASymphony DNA Midi kit (Qiagen). Fragmentation and indexing of the whole genome through tagmentation have been performed with Illumina DNA PCR-Free Prep, Illumina. Then, paired end 150 base pair sequencing by synthesis was done using an Illumina NovSeq6000. The bioinformatics analysis is conducted in trio with sequencing data of the parents and the proband with a pipeline based on the DNA pipeline implemented in the DRAGEN v3.10 Server (Illumina). Alignment is done with reference genome GRCh37/hg19. Variant annotation is carried out using Nirvana (Illumina), filtering and variant prioritization using Velona. An *in-silico* exome panel is applied and only variants within the target region of that panel are further analyzed.

**Supplemental references for in-silico tools used**

CADD Phred scores used were obtained from v1.7 ; Alphamissense scores were obtained from v2. SpliceAI scores were obtained from v3. SPiP scores were obtained from v2.

Rentzsch, P., Witten, D., Cooper, G. M., Shendure, J. & Kircher, M. CADD: predicting the deleteriousness of variants throughout the human genome. *Nucleic Acids Res.* **47**, D886–D894 (2019).

Cheng, J. *et al.* Accurate proteome-wide missense variant effect prediction with AlphaMissense. *Science* **381**, eadg7492 (2023).

Jaganathan, K. *et al.* Predicting Splicing from Primary Sequence with Deep Learning. *Cell* **176**, 535-548.e24 (2019).

Leman, R. *et al.* SPiP: Splicing Prediction Pipeline, a machine learning tool for massive detection of exonic and intronic variant effects on mRNA splicing. *Hum. Mutat.* **43**, 2308–2323 (2022).

Chm, R., Dev, P. & Db, A. DynaMut2: Assessing changes in stability and flexibility upon single and multiple point missense mutations. *Protein Sci. Publ. Protein Soc.* **30**, (2021).

Yariv, B. *et al.* Using evolutionary data to make sense of macromolecules with a ‘face-lifted’ ConSurf. *Protein Sci. Publ. Protein Soc.* **32**, e4582 (2023).

Sobreira, N., Schiettecatte, F., Valle, D. & Hamosh, A. GeneMatcher: a matching tool for connecting investigators with an interest in the same gene. *Hum. Mutat.* **36**, 928–930 (2015).

Baux, D. *et al.* MobiDetails: online DNA variants interpretation. *Eur. J. Hum. Genet. EJHG* **29**, 356–360 (2021).
